# Temporal dynamics of airborne fungi in Swedish forest nurseries

**DOI:** 10.1128/aem.01306-24

**Published:** 2025-01-16

**Authors:** Rebecca Larsson, Audrius Menkis, Åke Olson

**Affiliations:** 1 Department of Forest Mycology and Plant Pathology, Uppsala BioCenter, Swedish University of Agricultural Sciences 469025 , Uppsala, Sweden; Royal Botanic Gardens, Surrey, United Kingdom

**Keywords:** conifer seedlings, *Diplodia sapinea*, fungal pathogen, *Picea abies*, *Pinus sylvestris*, spore trap

## Abstract

In Sweden, reforestation of managed forests relies predominantly on planting nursery-produced tree seedlings. However, the intense production using containerized cultivation systems (e.g., high seedling density, irrigation from above, regular fertilization) creates favorable conditions for fungal infections. Despite the harmful role of diseases in forest nurseries, the origin and dispersal factors of fungal pathogens remain largely unknown. A better understanding of the airborne spread of pathogens could improve the prediction of fungal infection, ultimately optimizing preventative methods and decreasing the use of fungicides. This study investigated the temporal dynamics of airborne fungi in forest nurseries, with a focus on fungal pathogens. Airborne fungi were monitored in four Swedish forest nurseries over two growing seasons using spore traps and high-throughput sequencing. Fungal pathogens were identified using bioinformatics and quantified with quantitative PCR. Results showed strong temporal shifts of airborne fungal diversity and community composition following the growing seasons. The airborne spread included high abundances of important fungal pathogens (e.g., *Cladosporium* sp., *Botrytis cinerea*, *Alternaria* sp., *Sydowia polyspora*, and *Melampsora populnea*) with individual temporal and spatial variations. In general, the deposited spore loads of nursery pathogens correlated positively with increased temperature and negatively with higher precipitation. This was expressed the strongest for *Cladosporium* sp., *Alternaria* sp., and *M. populnea*, which suggests a higher availability of fungal inoculum in warm and dry periods. This study highlights the influence of seasonality on the temporal dynamics of economically important fungal pathogens in Swedish forest nurseries, which should be considered in the development of a local decision support system.

**IMPORTANCE:**

Fungal diseases in forest nurseries have significant environmental and economic impacts on the tree seedling production. This study highlights the role of seasonality in the airborne spread of fungal pathogens in Swedish forest nurseries. By analyzing airborne fungal spores using advanced sequencing and monitoring techniques, key fungal pathogens and their dispersal patterns over two growing seasons were identified. The findings indicate that warmer, drier periods may increase the spread of fungal pathogens, emphasizing the need for targeted preventative measures. Understanding these temporal dynamics can help optimize the use of fungicides in forest nurseries, thereby promoting more sustainable and environmentally friendly management practices. This research provides valuable insights for improving disease management in forest nurseries, ultimately supporting sustainable tree seedling production.

## INTRODUCTION

In Sweden, forests are important for providing several key ecosystem services, including the high economic value gained from wood and fiber production (1). To ensure sufficient regeneration after clear-cut harvesting, the forests are mainly (87%) reforested by planting nursery-produced tree seedlings (2). Therefore, the annual production of forest tree seedlings now exceeds 400 million, making Sweden the second-largest producer in Europe (3). The seedling production is mainly composed of *Picea abies* and *Pinus sylvestris*, while *Pinus contorta*, *Pseudotsuga menziesii*, *Larix* sp., and broadleaved tree seedlings are produced to a lower extent (2). The seedlings are densely grown using containerized cultivation systems elevated above the ground (4). The intense production process, ranging from seed germination in greenhouses to outdoor cultivation, may expose seedlings to stress and create favorable conditions for fungal infections. Fungal diseases can result in substantial losses in forest nurseries, making them economically significant. Critical factors include varied environmental conditions, high moisture levels enhanced by automatic irrigation from above, and intensive fertilization (4).

Fungal infections in forest nurseries are prevented by cultural control measures (e.g., removal of infected seedlings, the use of clean equipment, and good general hygiene), as well as chemical and biological control treatments (4). Systems capable of predicting the risk of fungal infections can be effective tools, enabling fungicide application only when there is a considerable risk of infection (5). Such methods have been successfully implemented in other systems (e.g., forecasting risks for potato late blight in potato [5] or Botrytis fruit rot in strawberries [6]). Still, preventing fungal infections by applying control measures based on decision support systems (e.g., disease forecasting systems) has not yet been implemented in forest nurseries.

Many fungal pathogens spread via spores (sexual or asexual) and other propagules, and their dispersion serves as the primary transmission of diseases. There are several fungal-specific factors that determine airborne dispersion (e.g., the production and release mechanism of spores, the size and shape of spores, and their dispersion distance) (7). Larger spores are suggested to more easily attach to a favorable substrate, while smaller spores would enable longer dispersal distances (8). The dispersal range can span from meters (e.g., among some ectomycorrhizal fungi, such as *Telephora americana* and *Lacaria laccata*) (9) to many kilometers (e.g., *Alternaria* spp.) (10). Since the frequency of spores declines with greater distance from the spore source (11), the deposition of airborne fungal spores is likely to be dominated by closely located sources (9). In addition to the dispersal distance, the spread of airborne fungal spores can be influenced by abiotic (e.g., wind, rain, humidity, and air temperature) and biotic (e.g., vegetation type) factors.

Both vegetation type and meteorological factors can significantly impact the composition of the airborne fungi (12, 13). The vegetation type plays a key role for the presences of specific fungal species. For example, the growing season of host plants seems to align with a dominance of spores produced by associated fungal pathogens (14, 15). In addition, the weather conditions will determine the spore release. *Botrytis* spp., which have a broad host range, have their spore release daily under favorable conditions (i.e., under warmer temperatures) (16). In contrast, *Sirococcus conigenus*, a pathogen recognized to infect both *Picea* spp. and *Pinus* spp., spread more locally in May and June under wet weather conditions (17). Temporal variations in fungal community composition can be driven by seasonality and variations in local weather conditions between and within observation years (12, 18). Thus, the deposited spore load attained from airborne fungal dispersal is likely influenced by changes in local weather conditions and shifts following the vegetation season. However, to acquire informative data on the deposition of airborne fungal communities, the appropriate sampling method should be considered (13).

Various studies have utilized diverse sampling methods for spore trapping to monitor the occurrence of airborne fungi, including active and passive sampling techniques (13, 18, 19). Active traps (e.g., volumetric air samplers) can give an indication of the potential spore deposition suspended in the air and are suggested to be better at capturing more of the long-distance dispersed spores (13, 15). In contrast, deploying passive spore traps (e.g., filter papers or funnel traps) can potentially monitor the realized spore deposition captured by gravity or rainfall (13, 20, 21). Therefore, passive spore traps will better represent the short distance and locally dispersed spore composition. In addition, passive spore traps are less expensive and more useful for studies that require multiple replicates (22).

Monitoring spore dispersion in forest nurseries during the outdoor period of seedling production has the potential to determine the relative importance of the surrounding environment and weather conditions (i.e., temperature fluctuations and variations in precipitation over time), which can create conducive conditions for fungal infection. In this study, we aimed to investigate the temporal dynamics of airborne fungal communities with a focus on fungal pathogens critical for forest nurseries (4, 23, 24). Fungal communities were monitored within four forest nurseries in different geographical regions in Sweden and over two growing seasons. We used DNA metabarcoding of spores from spore traps, as this method provides high-resolution data on airborne fungal diversity and the relative abundance of species (25). The interpretation of temporal shifts in fungal community structures was improved by incorporating quantitative polymerase chain reaction (qPCR) measurements to gain the absolute abundance of species (19, 26). Fungal pathogens known to the forest nurseries were further analyzed in response to the local weather condition (i.e., temperature and precipitation). The following hypotheses were tested: the fungal diversity and community composition will be influenced by seasonal changes following the seedling growth and by the nursery location and the sampling year. Furthermore, we expected the total spore load and the spore load of specific fungal pathogens to alter following shifts in temperature and precipitation over the growing season.

## MATERIALS AND METHODS

### Study sites and sampling

**Fig 1:**
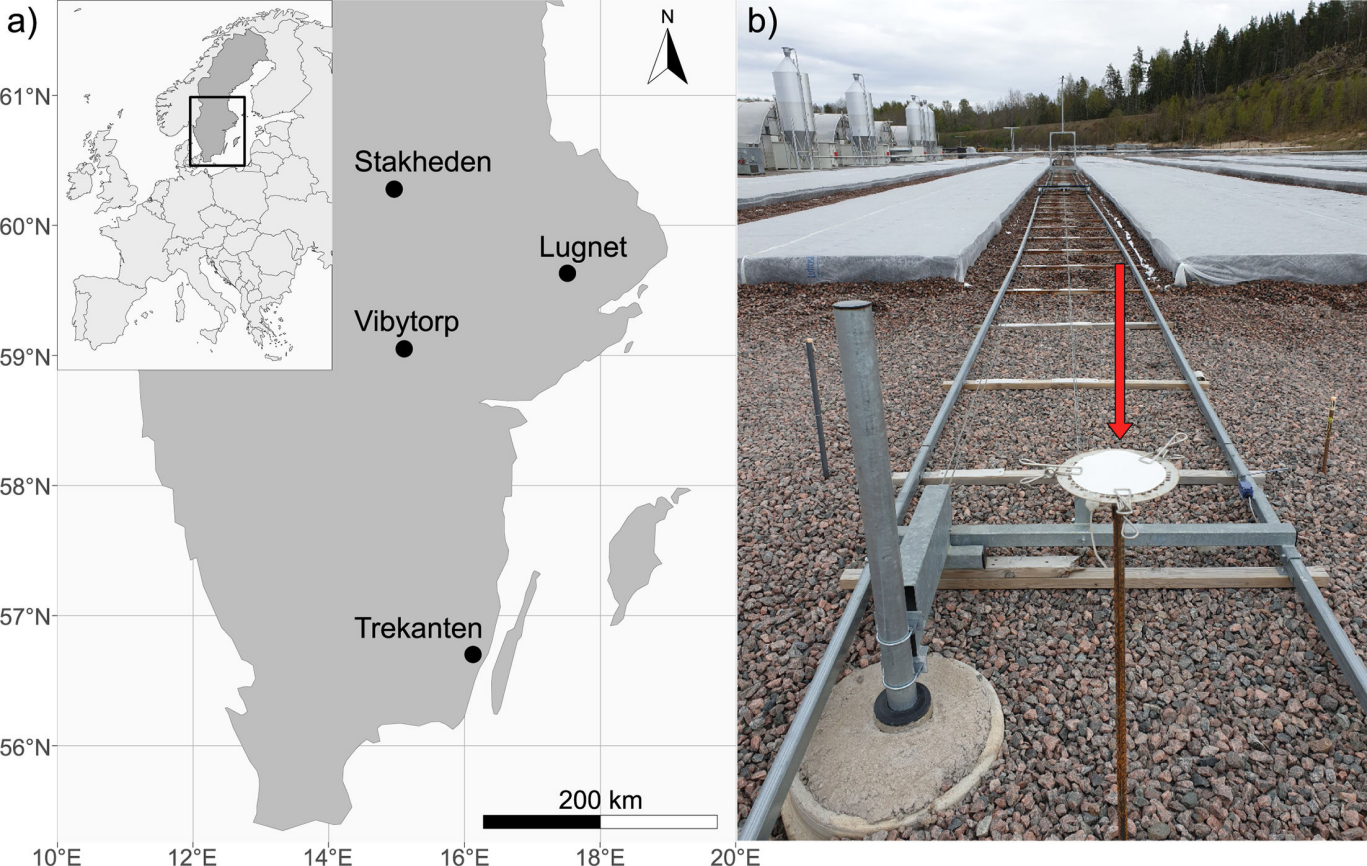
Sampling sites and spore trap used in the study. (**a**) Distribution of forest nurseries in Sweden and (**b**) the type of passive spore trap (arrowed) used in the study; seedlings are placed in rows and covered to be protected from frost damage during spring at Vibytorp nursery. The map was obtained using the r-package “rnaturalearth”.

Samples for this study were collected over two seedling-growing seasons (2020–2021) from week 18 in early May until week 46 at the end of November (Table S1). In the first season, samples were collected in three Swedish forest nurseries: Lugnet (59°37.9045′N, 17°30.8673′E), Vibytorp (59°3.0173′N, 15°6.6229′E), and Trekanten (56°37.9045′N, 16°7.4627′E), while the second season included an additional forest nursery: Stakheden (60°16.7138′N, 14°57.7879′E) (Fig. 1). The forest nurseries belonging to the forestry company, Sveaskog (business unit Svenska Skogsplantor), are in different regions of Sweden, and the areas surrounding these nurseries are of varying vegetation structures. At Stakheden nursery, the vegetation consists of mainly *P. sylvestris*-dominated forests, including individuals of *P. abies* and broadleaved tree species (e.g., *Betula* spp., *Populus tremula*, and *Sorbus aucuparia*) in the eastern, northern, and western sides of the nursery. In the south, the area consists of meadows and private gardens. The area around Lugnet nursery consists of mixed coniferous forests (*P. abies*/*P. sylvestris*) on the northern and western sides, while agricultural fields dominate the eastern side of the nursery. Along the southern side of the nursey, there is a lake shore with a belt of *P. sylvestris* and broadleaved trees. At Vibytorp nursery, the western and southern sides are covered by mixed coniferous (*P. abies*/*P. sylvestris*) and deciduous (e.g., *P. tremula* and *Betula* spp.) forests. The northern side consists of agricultural fields, and a small town populates the northeastern side. Trekanten nursery is mainly surrounded by agricultural fields with small patches of conifers (*P. abies*, *P. sylvestris*, and *Larix* spp.) and broadleaved tree species (e.g., *P. tremula*, *Salix* spp., *Quercus* spp., *Betula* spp., and *Fagus sylvatica*). A small town populates the southern and western sides.

Sensors for recording meteorological data (air temperature and precipitation) were used at each forest nursery. In cases when the sensors did not record data, missing data were obtained from a nearby weather station belonging to the Swedish Meteorological and Hydrological Institute (27). Meteorological data were recalculated into mean temperature, mean precipitation, and total accumulated precipitation for the days the filter papers were exposed to spore deposition, mostly 7 days ± 1 day. Total degree-day accumulation was calculated by summarizing daily temperatures (>0°C) from 1 January until each day of sampling.

To capture the locally realized airborne fungal community, three passive spore traps were used at each nursery and placed to cover the whole production area. At Stakheden and Lugnet nurseries, the traps were placed along a transect with one spore trap in the central part and two at each end of the nursery, 200–295 m between spore traps. At Vibytorp and Trekanten nurseries, the traps were placed in a triangle of 153–475 m between spore traps. Each spore trap was located ca. 5 meters from a field used to cultivate containerized conifer seedlings (Fig. 1). The spore traps consisted of one horizontally placed filter paper (90 mm diameter; Munktell, Ahlström, Sweden) attached using three metal clips on the top of a metal net platform ca. 1.2 m above the ground (Fig. 1) (21). To improve the trapping capacity, the filter paper was soaked in 4× TE buffer (40 mM Tris–HCl, 4 mM EDTA) and dried at 60°C for 48 h before being used in the field (19, 21). Filter papers were changed weekly using disposable gloves, collected in 50 mL Falcon tubes, and stored at −20°C until DNA extraction.

### DNA extraction, amplification, and sequencing

Filter papers were washed according to Zhang et al. (21) to extract spores from filters and assess fungal communities. To each Falcon tube containing a filter paper, 20 mL sodium dodecyl sulfate (SDS) buffer (50 mM Tris pH 8, 50 mM EDTA pH 8, 3% SDS, 1 M NaCl) was added, followed by incubation at 65°C in a water bath for 90 min. The tubes were inverted every 20 min during the incubation and vortexed at the end. After the filter paper had been removed, 20 mL of isopropanol was added to the SDS solution. The tubes were vortexed thoroughly and left to incubate at room temperature overnight. The following day, the tubes were centrifuged at 7,000 rpm for 10 min, and all, but 1–2 mL of the supernatant, was removed. The remaining supernatant was transferred into a 2 mL screw-cap tube containing a mixture of glass beads of varying sizes (ca. 200 mg 3 mm beads, ca. 200 mg 0.4 mm beads, ca. 130 mg 0.2 mm beads, ca. 4 mg diatomaceous earth) and further centrifuged at 16,200 ×*g* for 5 min. The remaining supernatant was removed, and the pellet was resuspended in 500 µL MC1 lysis buffer (NucleoMagPlant kit; Macherey-Nagel, Düren, Germany).

In total, DNA was extracted from 567 samples following the protocol of the NucleoMagPlant Kit (Macherey-Nagel, Düren, Germany). The samples were lysed at 5,000 rpm for 2 × 30 s using a Precellys 24 Tissue Homogenizer (Bertin Instruments, Montigny-le-Bretonneux, France), and DNA was extracted using the Maelstrom 4800 Extraction Robot (Taiwan Advanced Nanotech Inc., Taoyuan City, Taiwan). DNA concentrations were not determined, as the concentrations were too low to be detectable using a NanoDrop One spectrophotometer (Thermo Scientific, Rodchester, NY, USA). All samples were instead diluted 1:10, and the ITS2 rDNA region was amplified by PCR using the fITS7 (28) and ITS4 (29) primers tagged with unique identifier sequences (Table 1).

**TABLE 1:** List of unique identifier tags (8 and 1 bp linkers) attached to the primer sequences fITS7 and ITS4 used to amplify the ITS2 rDNA region

ID	Tags	ID	Tags	ID	Tags
tag1	CACACGATC	tag38	CATGTACTG	tag76	CGTGTAGTC
tag4	CACATAGTC	tag39	CATGTCGCT	tag77	CGTGTGTAC
tag5	CACATGACT	tag41	CCACGTCAC	tag78	CTACATGAG
tag6	CACGATCAG	tag42	CCACTATCG	tag79	CTACTGATC
tag7	CACGTGCTC	tag44	CCAGATACT	tag80	CTAGAGCAC
tag8	CACTATAGC	tag45	CCAGCGTAG	tag81	CTAGCTATC
tag9	CACTATGTG	tag46	CCAGTATGT	tag82	CTAGTCATG
tag10	CACTCAGAG	tag47	CCATGTAGT	tag83	CTATAGCTG
tag11	CACTCTCAC	tag48	CCGACTGAT	tag84	CTATATCGC
tag12	CACTGCTAC	tag49	CCGAGCACT	tag85	CTATCACTC
tag13	CAGACAGTG	tag50	CCGAGTGTC	tag86	CTATCTCAG
tag14	CAGACATCT	tag51	CCGATAGAC	tag87	CTATCTGCT
tag15	CAGAGACGC	tag52	CCGCATCGT	tag88	CTATGCTAG
tag16	CAGAGCTCG	tag53	CCGTAGCAT	tag89	CTATGTGTG
tag17	CAGAGTATG	tag54	CCGTATATG	tag90	CTCACTAGC
tag18	CAGATACAG	tag55	CCGTGTCAG	tag91	CTCACTCAT
tag20	CAGCACTAT	tag56	CCTACATCG	tag92	CTCATAGAG
tag21	CAGCGATAC	tag57	CCTACGCTC	tag93	CTCATCAGT
tag22	CAGCTAGAT	tag58	CCTAGACTG	tag94	CTCATCTCG
tag23	CAGCTCACT	tag60	CCTCGAGTC	tag95	CTCGCACTG
tag24	CAGTATCTC	tag61	CCTCTCTGT	tag96	CTCGCAGAT
tag25	CAGTGCAGT	tag63	CCTGTATAG	tag97	CTCTAGATC
tag26	CAGTGTGAT	tag65	CGACTAGCT	tag98	CTCTATGAC
tag28	CATAGTCTC	tag66	CGAGTAGAG	tag100	CTCTGACAC
tag29	CATAGTGAG	tag67	CGCGTCTAT	tag101	CTCTGCGAT
tag30	CATATGTCG	tag68	CGCTATACG	tag104	CTGAGAGAT
tag31	CATCGACAG	tag69	CGTACAGAT	tag105	CTGAGCTGT
tag32	CATCGCTCT	tag70	CGTACTCAC	tag106	CTGAGTCAC
tag33	CATCTACGC	tag71	CGTAGCGTC	tag107	CTGATACTC
tag34	CATCTCGTG	tag72	CGTATCGCG	tag108	CTGATCGTG
tag35	CATCTCTAC	tag73	CGTCAGTCT	tag109	CTGATCTAC
tag36	CATGATACG	tag74	CGTCGCTAG	tag112	CTGCTATGT
tag37	CATGCGTCT	tag75	CGTCGTGTG	tag113	CTGCTGACG

PCR reactions and cycling program followed Larsson et al. (23) using an optimized number of PCR cycles. In case a sample did not meet the criteria for a satisfied PCR product, the sample was further diluted into either 1:20 or 1:40 concentrations until an optimal number of PCR cycles was met. PCR products were cleaned using the AMPure Kit (Beckman Coulter, Indianapolis, IN, USA), and DNA concentration was quantified using a Qubit fluorometer (Thermo Fisher Scientific, MA, USA). An equivalent molar mix of purified PCR products was pooled into seven pools. A sample containing non-ITS sequences of *Heterobasidion irregulare* (a species currently not present in Sweden and not expected to be detected among the samples) of different lengths was added to each pool to account for possible sequence length biases between amplification products. The E.Z.N.A. Omega Cycle Pure Kit (Omega Biotek, Norcross, GA, USA) was used to clean the pooled libraries and assessed in BioAnalyser DNA 7500 (Alignment Technologies, Boulder, CO, USA) to control amplicon quality and size distribution. Finally, pooled libraries were sequenced on the PacBio RSII platform using seven SMRT cells (one per library) by SciLifeLab NGI (Uppsala, Sweden).

### Quantification of fungal DNA from filter paper

To assess the abundance of the total fungal DNA, the ITS2 rDNA gene was used for qPCR. Prior to the qPCR assays, potential PCR inhibition was tested by running all samples diluted 1:5 and spiked with a circular pGEM plasmid (Promega, WI, USA) with plasmid-specific primers (T7 and SP6). Amplification of the samples was compared to water controls, and no inhibition was present in 580 samples, while 15 samples were further diluted until no inhibition was present in the samples. The inhibition test was used to decide on the final dilution of the DNA template, which, for most samples, was 1:5. Each reaction contained 2 µL template DNA (concentration of <1 ng), 1× iQ SYBR Green Supermix (BioRad, CA, USA), forward primer (fITS7) concentration of 0.5 µM, and reverse primer (ITS4) concentration of 0.3 µM in a total volume of 15 µL. The PCR cycling program was set to 95°C for 3 min, followed by 34 cycles of 95°C for 15 s, 57°C for 30 s, 72°C for 30 s, and 78°C for 5 s using the BioRad CFX Connect Real-Time System (BioRad Laboratories, CA, USA). The program ended with 95°C for 15 s, 65°C for 5 s, and 95°C for 5 min. Standard curves were obtained by serial dilutions of linearized plasmid fragments of the ITS gene from 10^8^ to 10^2^ copies µL^−1^. The total ITS copy number per sample was calculated as the mean of two technical replicates according to the qPCR result using the standard curves. Two samples were not included in the qPCR assay due to the lack of DNA templates, and 12 samples did not yield measurable concentrations after the qPCR.

### Bioinformatics

The quality filtering and clustering of sequences were done through the SCATA pipeline (30). During the filtration step, sequences characterized by low quality were excluded. This encompassed sequences that were shorter than 200 base pairs, exhibited poor read quality (Q < 20), lacked either a sample tag or a primer, or contained primer dimers. Homopolymers of the sequences were collapsed into three base pairs. Following the quality filtration, the sequences underwent clustering into operational taxonomic units (OTUs) through single linkage clustering, with a minimum similarity threshold of 98.5% (31). The OTUs containing less than 10 reads and appearing in less than two samples were removed from further analysis. The massBLASTer (UNITE/INSD fungi) databases implemented in the PlutoF biodiversity platform were used to classify the fungal OTUs taxonomically and separate non-fungal OTUs from the data set (32). The OTUs identified from the massBLASTer output were kept only if the sequence similarity exceeded 80%, ensuring a reliable identification at the phylum level. Any OTUs displaying a similarity below 80% were categorized as “non-fungal” and consequently excluded from subsequent analyses (24, 33).

Fungal taxonomies were assigned manually for the 200 most common fungal OTUs (represented by 88% of the sequence reads), while the remaining fungal OTUs (12%) were set as unassigned. The criteria for genus-level identification were set to a minimum sequence-similarity threshold of 94%, while taxon-level identification required a sequence similarity equaling or exceeding 98%. Fungal OTUs with high matches to multiple species were ascribed to their common genus. Using the FungalTraits database (34), primary lifestyles were assigned to fungal OTUs taxonomically classified at the genus or higher level, revealing 13 primary lifestyles among the 200 most common fungal OTUs. The primary lifestyles were grouped into plant pathogen, saprotroph (litter, nectar/tap, soil, wood, unspecified), parasite (animal, lichen), mycoparasite, lichenized, epiphyte, endophyte, and ectomycorrhizal. Groups represented by less than 3% of the fungal OTUs were further grouped into “others,” and fungal OTUs not assigned to a lifestyle were set as “unassigned.”

Following the assignment of primary lifestyles and the taxonomic classification of fungal OTUs, fungal pathogens with significance for forest trees or seedling production were selected based on previous reports from forest nurseries (4, 23, 24, 35, 36). In total, eight fungal OTUs (hereafter referred to as “nursery pathogens”) were further analyzed for their occurrence and distribution in the forest nurseries based on their spore loads (ITS copy number) obtained from each sample.

### Statistical analysis

All statistical analyses were performed using R Statistical Software v 4.3.0 and RStudio (37, 38), and all graphs were illustrated using the R package ggplot2 (39). Rarefaction curves (Fig. S1) were generated prior to statistical analyses as a quality control for the sequencing depth of each sample using the Vegan package (40).

The deposited spore load was analyzed using the quantified ITS copy number per sample. The spore load was estimated by first calculating the relative abundance on non-rarefied data sets, followed by multiplying the relative abundance with the measured ITS copy number obtained from the qPCR analysis. The correlation between ITS copy number and weather parameters (mean temperature, mean precipitation, and accumulated temperature) was tested on Box–Cox transformed data sets to improve the distribution of residuals using linear models and correlation tests using Pearson’s method. The effect of year, nursery location, week, and the interaction between year and week on the total spore load (ITS copy number) and the spore load of selected nursery pathogens was tested by constructing general linear mixed-effects models adjusted for unbalanced data set. A Box–Cox transformation of the data set was used to improve the distribution of residuals within the models. Group differences were assessed through pairwise comparisons on estimated marginal means in the R package Emmeans v. 1.7.4.1 (41).

The number of fungal OTUs, the Shannon diversity index, and the Simpson’s evenness index were used to assess the diversity of the airborne fungal communities (42). The effect of year, nursery location, week, and the interaction between year and week on fungal diversity was tested by constructing general linear mixed-effects models adjusted for unbalanced data sets. The square root of the total number of reads for each sample was used as the first factor in the models to account for variance in sequencing depth among samples (31, 43), and spore trap ID was used as a random factor in the models. The distribution of residuals and Q–Q plots were used to check for normality of the data which was, if necessary, transformed using a Box–Cox transformation to improve the distribution of residuals within the models. Group differences were analyzed using pairwise comparisons on estimated marginal means in the R package Emmeans v. 1.7.4.1 (41).

The fungal community composition was analyzed using the Phyloseq and Vegan packages (40, 43). The permutational multivariate analysis of variance (PERMANOVA) was applied to the Bray–Curtis dissimilarity matrix, utilizing 999 permutations, for subsequent analyses of the group effects of year, nursery location, week, and the interaction of year and week on the fungal community composition. To assess the assumption of PERMANOVA, a permutational analysis of multivariate dispersion was conducted to test for the multivariate homogeneity of group dispersion (variance) in species composition between years and between nursery locations for each year (44). The square root of the total number of reads for each sample was used as the initial factor in each model, aiming to address variance attributed to sequencing depth variations across samples (31, 43). Fungal community composition was analyzed using Hellinger-transformed relative abundance tables, and group differences were assessed through pairwise comparisons using the R package Adonis v. 0.4 (45). Principal coordinate analysis based on the Bray–Curtis dissimilarity matrix was used to create ordination plots presenting the variation in fungal community composition.

## RESULTS

### Variation of deposited airborne spores across weeks, nurseries, and years

**Fig 2:**
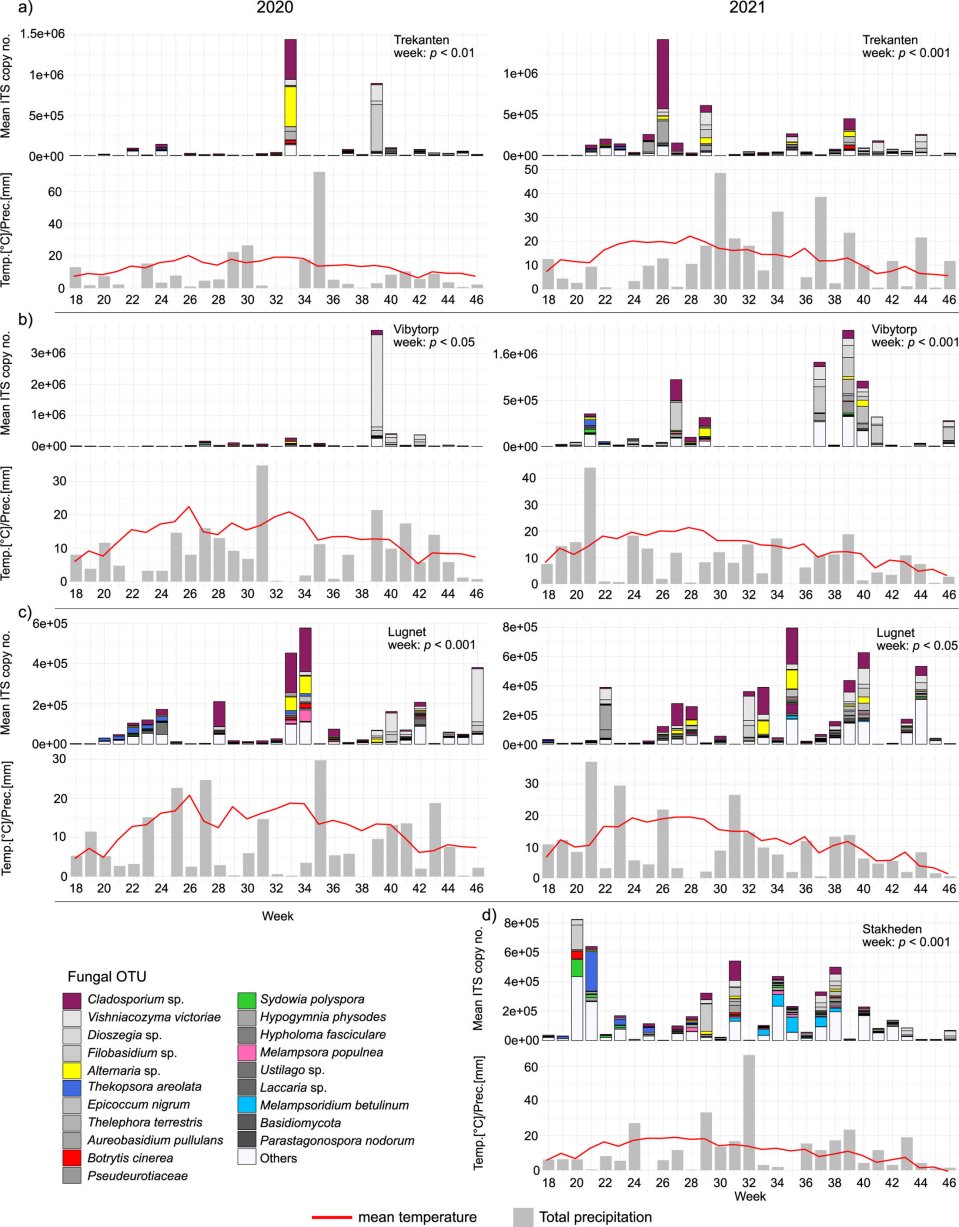
Seasonal weather data and the mean ITS copy number and abundance of the 20 most common fungal OTUs obtained from spore traps per week in (**a**) Trekanten, (**b**) Vibytorp, (**c**) Lugnet, and (**d**) Stakheden nurseries in 2020 and 2021. Remaining fungal OTUs are grouped into “others.” Fungal pathogens of significance for forest trees and seedlings are highlighted using different colors.

Deposited airborne spores were obtained from 567 samples collected from four forest nurseries over two growing seasons. The spore load measured as the ITS copy number varied between years and over the weeks of spore collection (Table 2). A pairwise comparison showed that the overall ITS copy number was higher in 2021 than in 2020 (*P* < 0.001). Further analyses within each nursery revealed similar results of higher ITS copy numbers in 2021 at both Trekanten (*P* < 0.01) and Vibytorp (*P* < 0.01) nurseries (Fig. 2). However, no differences were found between years at Lugnet nursery (*P* > 0.05). Furthermore, the ITS copy number was found to vary between weeks within each nursery for each year (Fig. 2, *P* < 0.05). The spore load peaked with >10^6^ ITS copies on one or two occasions during the growing seasons at Trekanten (w 33 in 2020 and w 26 in 2021) and Vibytorp (w 39 in 2020 and w 37 and 39 in 2021) nurseries, whereas spore loads from Lugnet (w 35 in 2021) and Stakheden (w 20 in 2021) nurseries peaked around 8 × 10^5^ ITS copies (Fig. 2). However, the nursery location did not impact the overall spore load (Table 2, *P* > 0.05).

**TABLE 2:** Effects of year, geographical location (nursery), week, and the interaction of year and week on the fungal ITS copy number^*a*^

	Chisq	df	Pr (>Chisq)
(Intercept)	2751.6	1	<0.001***
Year	20.9	1	<0.001***
Nursery	2.9	3	0.40
Week	94.8	28	<0.001***
Year: Week	50.2	28	0.01**

^*a*^ Analyses are based on datasets adjusted using a Box–Cox transformation in general mixed-effects models. Significant values are indicated with *(*P* < 0.05), **(*P* < 0.01), and ***(*P* < 0.001).

The overall spore load was moderately positively correlated with the accumulated temperature at Vibytorp (*P* < 0.05) and Lugnet (*P* < 0.05) nurseries (Fig. S2). As regards the accumulated temperature, Trekanten nursery showed a positive, but non-significant correlation (*P* > 0.05), while Stakheden showed a negative, but non-significant correlation to accumulated temperature (*P* > 0.05).

### Fungal diversity and community composition of deposited spores in forest nurseries

In total, 1,277,473 high-quality sequences were generated and clustered into 5502 global clusters (OTUs) and 5233 singletons, which were excluded from further analysis. Following further filtering (removal of “non-fungal” OTUs and fungal OTUs containing <10 reads and represented by less than two samples), the data set included 2,152 fungal OTUs represented by 1,067,884 sequences. The 200 most common fungal OTUs were represented by 944,886 (88.5%) sequences and belonged to either Basidiomycota (50.0%), Ascomycota (49.5%), or Chytridiomycota (0.5%) (Table S2). They were taxonomically classified to either species (44.5%), genus (33.5%), family (5.0%), order (3.5%), class (4.5%), or phylum level (9.0%) (Table S2).

**Fig 3:**
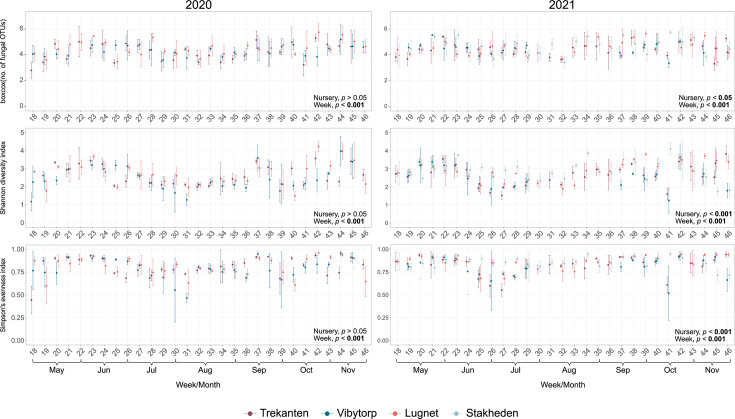
Fluctuation of the number of fungal OTUs, the Shannon diversity index, and the Simpson’s evenness index of fungal communities obtained from spore traps at Trekanten, Vibytorp, Lugnet, and Stakheden nurseries in 2020 and 2021. The mean values with error bars are indicated with points and the nurseries using different colors. Significant differences are indicated in bold.

The fungal diversity obtained from spore traps was estimated using the number of fungal OTUs, the Shannon diversity index, and the Simpson’s evenness index. The Shannon diversity index significantly differed between the nursery locations, the weeks, and the interaction between the years and the weeks (Fig. 3; Table S3, *P* < 0.001), while no difference was found between years (Table S3, *P* > 0.05). In contrast, the number of fungal OTUs and the Simpson’s evenness index showed significant differences between all tested factors (year, nursery, and weeks) (Table S3, *P* < 0.05). The fungal diversity was generally higher in 2021 than in 2020, as indicated by higher values of both Shannon diversity index and Simpson’s evenness index (pairwise comparison, *P* < 0.05). Further downstream analyses showed significantly different fungal diversities between the nursery locations in the year 2021 (Fig. 3; Table S4, *P* < 0.05), but not in the year 2020 (Fig. 3; Table S4, *P* > 0.05).

Pairwise comparisons resulted in a higher Shannon diversity index and Simpson’s evenness index in Stakheden and Lugnet nurseries than in Vibytorp nursery during the year 2021 (Table S5, *P* < 0.05). No differences between nurseries were found for the number of fungal OTUs (Table S5, *P* > 0.05). The sampling point (week) influenced the fungal diversity throughout the growing seasons, including all three diversity measures (number of fungal OTUs, *P* < 0.001; Shannon diversity index, *P* < 0.001; Simpson’s evenness index, *P* < 0.01). The fungal diversity changed over the season and was lowest during the summer and the difference between nurseries increased toward the end of the season (Fig. 3).

**Fig 4:**
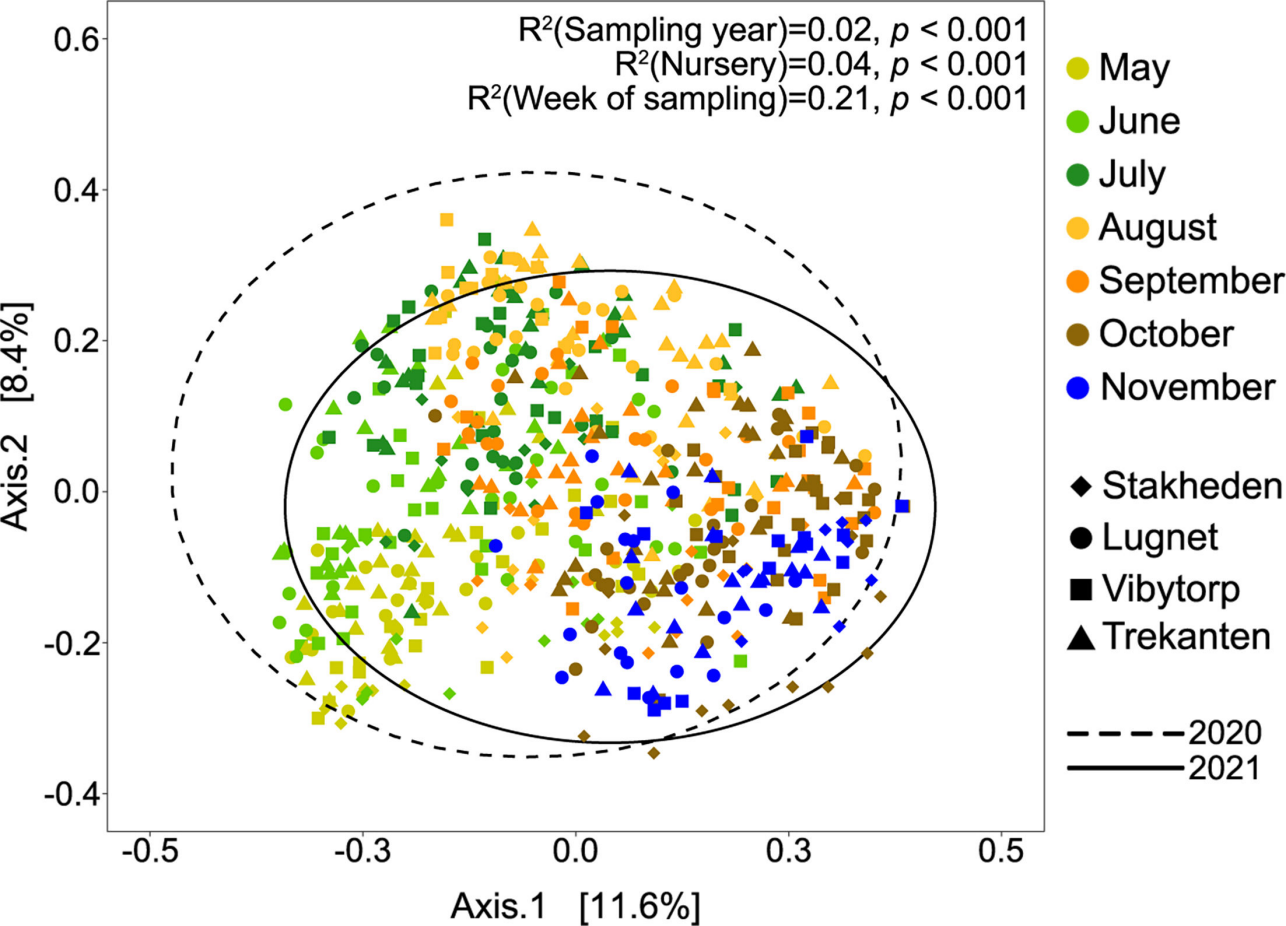
Principal coordinate analysis plot of fungal community sampled from spore traps at four forest nurseries over 2 years. Forest nurseries are indicated using different symbols, and different colors indicate the spore collection’s month (weeks combined). The ellipses represent a 95% CI around the group centroids of the year of spore collection and the percentage of variance explained by the x- and y-axes is presented in the brackets.

The fungal community composition of spore deposition was determined by seasonal changes rather than by location or year (Fig. 4). A PERMANOVA analysis showed that the week of spore trapping explained 21.2% of the variation (*P* < 0.001), while nursery location explained 3.5% (*P* < 0.001) and years 1.9% (*P* < 0.001) of the variation (Fig. 4; Table S6). Additionally, there was an interaction between the year and the week of spore dispersion, explaining 8.3% (*P* < 0.001) of the variation (Table S6). Differences in sequencing depth among the samples explained 0.4% (*P* < 0.001) of the variation.

### Occurrence and distribution of nursery fungal pathogens

The primary lifestyles of fungal OTUs obtained from spore traps were either saprotroph (38.5%), plant pathogens (28.0%), ectomycorrhizal (4.5%), lichenized (2.0%), parasite (2.0%), epiphyte (1.0%), mycoparasite (0.5%), or endophyte (0.5%) or were not taxonomically classified to be assigned a lifestyle (23.0%) (Table S2). There were no site-specific species detected, and the 20 most common fungal OTUs were detected in all forest nurseries (Table 3). *Cladosporium* sp. was the most common fungal OTU (20.2%) and was assigned by FungalTraits with saprotroph as the primary lifestyle (Table 3). However, *Cladosporium* sp. was also given a plant pathogenic guild (Table S2) and observed to infect conifer seedlings in Swedish forest nurseries (personal observations). Among the most common fungal OTUs with primary lifestyles as plant pathogens were *Thekopsora areolata* (4.3%), *Alternaria* sp. (4.2%), *B. cinerea* (1.9%), *S. polyspora* (1.5%), *Melampsora populnea* (1.1%), and *Melampsoridium betulinum* (0.8%) (Table 3).

**TABLE 3:** Relative abundance of the 20 most common fungal OTUs obtained from spore traps at Trekanten, Vibytorp, Lugnet, and Stakheden forest nurseries^*a*^

OTU	Phylum	Taxon	Lifestyle	Reference	Similarity	Relative abundance (%)				
						Trekanten	Vibytorp	Lugnet	Stakheden	All
5647_0	Ascomycota	*Cladosporium* sp.	Saprotroph	UDB0799152	243/243 (100%)	22.67	20.79	22.83	9.16	20.17
5647_1	Basidiomycota	*Vishniacozyma victoriae*	Saprotroph	OQ066334	234/234 (100%)	9.18	8.00	10.12	9.06	9.14
5647_3	Basidiomycota	*Dioszegia* sp.	Saprotroph	MN077496	220/220 (100%)	6.27	6.23	4.32	7.01	5.80
5647_2	Basidiomycota	*Filobasidium* sp.	Saprotroph	OQ448474	336/336 (100%)	2.21	12.47	3.33	9.83	6.28
5647_5	Ascomycota	*Alternaria* sp.	Plant pathogen	OP699763	253/253 (100%)	5.16	4.22	4.97	1.68	4.33
5647_4	Basidiomycota	*Thekopsora areolata*	Plant pathogen	OL471672	311/311 (100%)	3.76	4.06	4.25	5.33	4.22
5647_7	Ascomycota	*Epicoccum nigrum*	Plant pathogen	OR245533	249/249 (100%)	4.46	2.75	3.64	2.45	3.48
5647_8	Ascomycota	*Aureobasidium pullulans*	Saprotroph	UDB035654	249/249 (100%)	3.03	1.78	2.82	2.43	2.56
5647_6	Basidiomycota	*Thelephora terrestris*	Ectomycorrhizal	UDB0798946	313/313 (100%)	4.13	1.38	1.21	0.99	2.10
5647_11	Ascomycota	*Botrytis cinerea*	Plant pathogen	UDB035382	240/240 (100%)	2.58	1.76	1.95	0.94	1.94
5647_12	Ascomycota	Pseudeurotiaceae	Unassigned	OV989365	239/240 (99.6%)	1.14	1.70	2.13	1.97	1.70
5647_10	Ascomycota	*Sydowia polyspora*	Plant pathogen	OR069590	256/256 (100%)	1.02	1.94	1.10	2.52	1.50
5647_14	Ascomycota	*Hypogymnia physodes*	Lichenised	MK812139	247/247 (100%)	0.98	0.67	1.22	2.28	1.17
5647_9	Basidiomycota	*Melampsora populnea*	Plant pathogen	MT759632	325/325 (100%)	0.69	1.02	1.08	1.82	1.06
5647_13	Basidiomycota	*Ustilago* sp.	Plant pathogen	MH855347	386/386 (100%)	0.19	0.25	2.81	0.28	0.99
5647_19	Basidiomycota	*Hypholoma fasciculare*	Saprotroph	UDB0778345	304/304 (100%)	1.46	0.41	0.66	1.08	0.90
5647_16	Basidiomycota	*Melampsoridium betulinum*	Plant pathogen	MZ159583	313/313 (100%)	0.12	0.11	0.19	4.18	0.76
5647_17	Ascomycota	*Parastagonospora nodorum*	Plant pathogen	OW988187	247/247 (100%)	0.41	0.92	0.67	0.21	0.58
5647_21	Ascomycota	*Stemphylium vesicarium*	Plant pathogen	UDB035339	253/253 (100%)	0.97	0.17	0.48	0.06	0.49
5647_22	Basidiomycota	*Sporobolomyces roseus*	Mycoparasite	OQ066835	300/300 (100%)	0.37	0.71	0.65	0.10	0.50
Total of 20 fungal OTUs						70.79	71.33	70.43	63.38	69.68

^*a*^ The similarity column compares base pairs between the query and reference sequences from the GenBank database, with sequence similarity expressed as a percentage.

**Fig 5:**
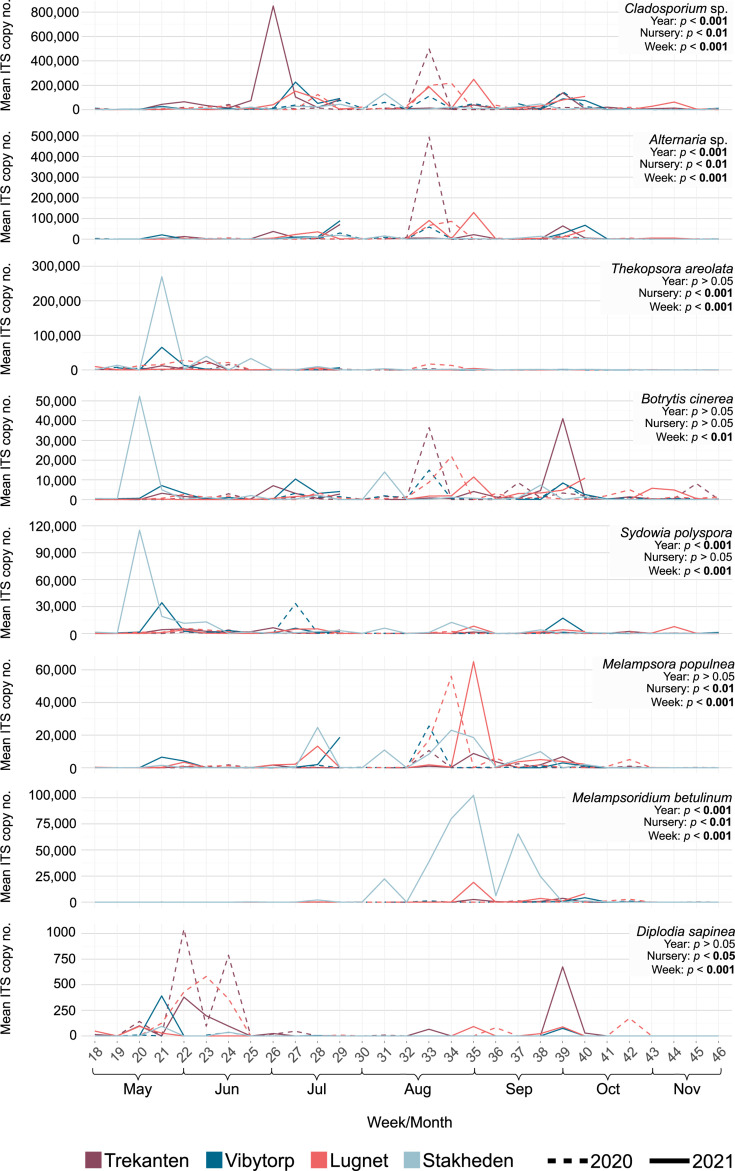
Temporal abundance expressed as the mean ITS copy number of significant nursery pathogens at Trekanten, Vibytorp, Lugnet, and Stakheden forest nurseries. Forest nurseries are indicated using different colors and years using dashed or solid lines. Significant differences are indicated in bold.

Nursery pathogens significant for forest trees or seedling production were selected, and their occurrence (in terms of ITS copy number) was further investigated. In addition to the more abundant pathogens (*Cladosporium* sp., *Alternaria* sp., *T. areolata*, *B. cinerea*, *S. polyspora*, *M. populnea*, and *M. betulinum*), spore deposition of *D. sapinea* was detected in the nurseries and included in the analyses (Table S2). The nursery pathogens were affected differently by nursery locations and years (Fig. 5; Table S7). In general, the deposited spores from the pathogens were similar between the nurseries. However, *T. areolata* and *M. populnea* had higher spore loads at Lugnet and Stakheden nurseries (Tables S8 and S9), whereas *Cladosporium* sp. and *Alternaria* sp. had lowest spore loads at Stakheden nursery (Table S9). The time point of spore collection during the growing season had the strongest effect on deposited spore loads of all nursery pathogens (Fig. 5; Table S7).

**Fig 6:**
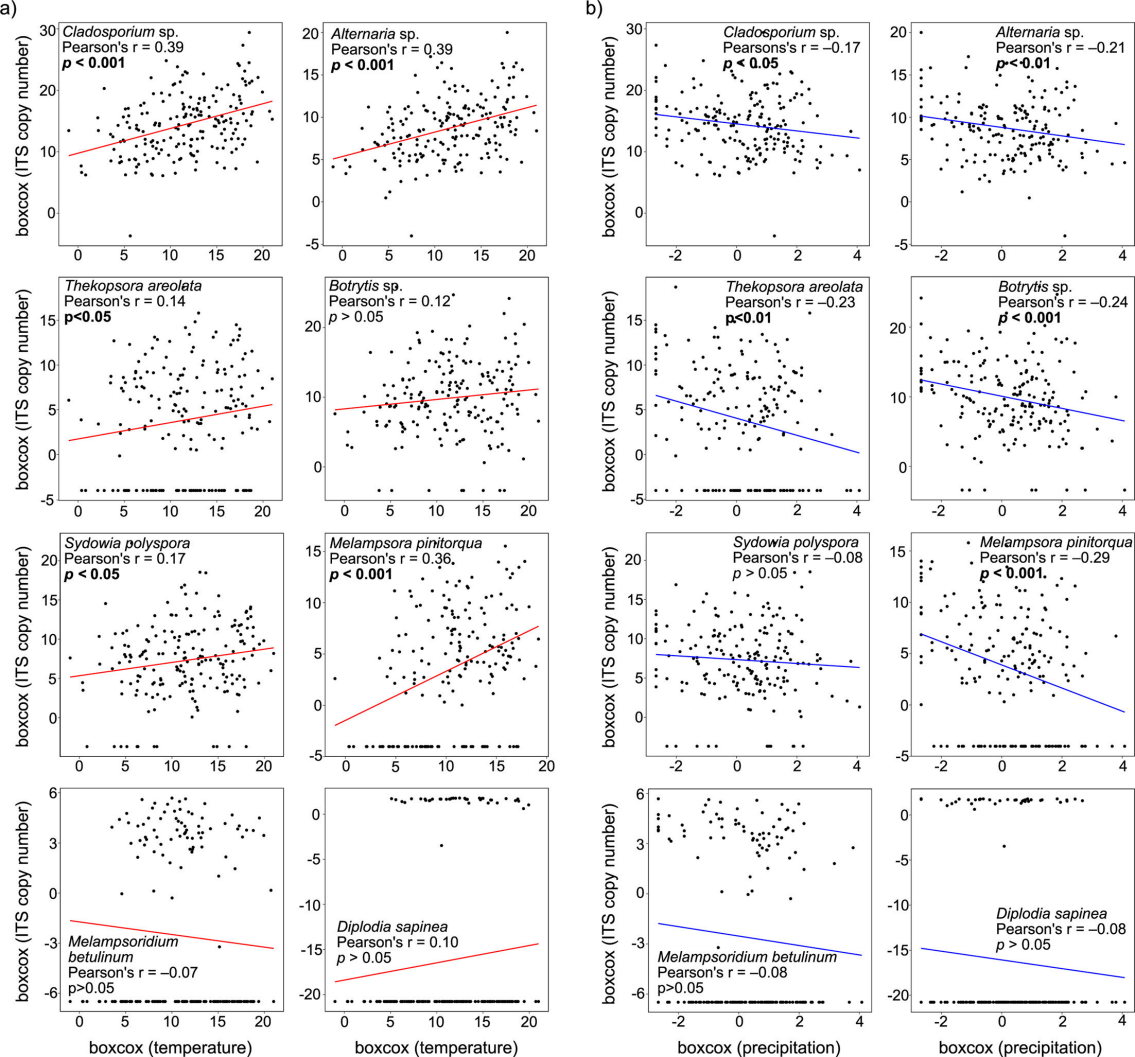
Correlation of ITS copy number obtained from spores of significant nursery pathogens to (**a**) temperature and (**b**) precipitation. Temperature and precipitation are presented on a relative scale using a Box–Cox transformation. Correlation factors are given as Pearson’s *r*, and significant differences are indicated in bold.

The spore loads of *Cladosporium* sp., *Alternaria* sp., and *M. populnea* were found to have a moderate positive correlation with temperatures (Fig. 6, *P* < 0.001), while *T. areolata* and *S. polyspora* showed weak positive correlations (Fig. 6, *P* < 0.05). However, the spore load of *B. cinerea*, *M. betulinum*, and *D. sapinea* did not correlate significantly with temperature (Fig. 6, *P* > 0.05). In addition, *M. populnea* showed a negative correlation with increased precipitation (Fig. 6, *P* < 0.001), while *Cladosporium* sp., *Alternaria* sp., *T. areolata,* and *B. cinerea* showed weak correlations with precipitation (Fig. 6, *P* < 0.05). *Sydowia polyspora*, *M. betulinum*, and *D. sapinea* did not significantly correlate with precipitation (Fig. 6, *P* > 0.05).

## DISCUSSION

Knowledge on the airborne dispersal of fungi in forest nurseries is still limited, and a better understanding would improve methods to prevent fungal infection of nursery-grown tree seedlings. This study investigated the temporal dynamics of airborne fungal communities and fungal pathogens from four geographically separated forest nurseries over two growing seasons using high-throughput sequencing. Results showed that temporal shifts of airborne fungal community composition and changes in species diversity were strongest within the growing seasons. High abundances of significant nursery pathogens were detected, and their occurrence varied over the growing seasons and between the forest nurseries.

### Temporal and spatial dynamics of the airborne fungal community

Temporal fluctuations and seasonal shifts of airborne microbial communities have consistently been reported and explained by, for example, shifting local vegetation structure (46), microclimate (12), or the origin of air mass (47). In this study, the temporal effect on the airborne fungal communities was expressed by differences in the fungal diversity between years and throughout the growing seasons. The week of sampling explained the biggest part (21.2%) of the variation, which suggests that the airborne fungal community composition in the forest nurseries was mainly influenced by the temporal shifts within the growing seasons. This is in accordance with the findings of Larsson et al. (23) on foliar fungal communities obtained from nursery-grown *P. sylvestris* seedlings. The interaction of year and week explained about half of the variation of community composition compared to the week, showing a combined effect of seasonal variation within and between years. In addition, the influence of temporal dynamics on the airborne spread of fungi was observed by the fluctuations of the overall deposited spore load, which also differed between years. The spore load of fungal communities could have both a positive (20) or negative (48) correlation with precipitation. However, this study did not find any significant correlation with either temperature or precipitation, which could be an indication of varying responses of airborne fungal community to weather conditions, resulting in the unclear overall trend for the total amount of spores in the air.

Temporal shifts changing the airborne fungal community structure are well-known events from both landscape (49) and local scales (20). However, a large part of the variation of the fungal community composition was not explained by any of the tested factors in this study, indicating that there might be other factors shaping the airborne fungal communities. For example, specific environmental factors (e.g., solar radiation, soil temperature, relative humidity, and soil moisture) can strongly influence the airborne fungal community composition (50), which could be contributing factors in the present study.

The spatial effect (nursery location) explained a considerably small part (five times less than the temporal variation explained by week) of the fungal community variation. Nevertheless, a higher fungal diversity was observed in the northern nurseries. This result could reflect the effect of a more heterogeneous landscape in the vicinity of the nurseries in the north of Sweden, as suggested by Karlsson et al. (51). However, the vegetation of the nearby surroundings, in combination with the local meteorological conditions, has been reported as a strong indicator of the airborne microbial community structure (52). Thus, the spatial effect on the fungal communities was expected to be larger than observed in this study. The lower spatial differences observed between the forest nurseries could indicate similar structures within and near the nursery locations (e.g., management practices and the large-scale production systems), and the temporal changes throughout the growing season were more important in shaping the airborne fungal community structures in the forest nurseries.

### Airborne nursery pathogens

Airborne fungi mainly belonged to Basidiomycota or Ascomycota, which was similar to recent reports of forest nursery-associated fungal communities (23, 24). The fungal OTUs commonly showed high identification matches, often at the species or genus level, implying that a significant portion of the detected fungi corresponds to widely recognized and thoroughly studied species and/or genera. The primary lifestyles were mostly saprotrophs and plant pathogens, and, to a lesser extent, ectomycorrhizal, lichenized, parasite, mycoparasite, epiphyte, or endophyte. Among the observed plant pathogenic species, eight fungal OTUs were previously shown to be forest tree and/or seedling pathogens (*Cladosporium* sp., *Alternaria* sp., *T. areolata*, *B. cinerea*, *S. polyspora*, *M. populnea*, *M. betulinum*, and *D. sapinea*) (4, 23, 24, 35, 36).

*Botrytis cinerea* is one of the most commonly known forest nursery pathogens in Northern Europe and can rapidly reproduce and spread under appropriate environmental conditions (4, 23, 36, 53, 54). In this study, *B. cinerea* occurred regularly among the nurseries but showed local differences in deposited spore loads. The deposition of *B. cinerea* spores was significantly negatively correlated with precipitation and positively, not significantly, correlated with the mean temperature. Previous reports of the airborne dispersal of *B. cinerea* include similar negative correlations with mean air humidity or precipitation and positive correlations with mean temperature (55) but also contrary results (56, 57). Context-dependent factors (e.g., geographical location or crop production system) can be essential in predicting the airborne occurrence of *B. cinerea*, and this study provides the first insight into airborne distribution in Swedish forest nurseries.

*Cladosporium* sp. and *Alternaria* sp. are known nursery pathogens that can cause infection and seedling loss in forest nurseries (23, 24, 33). In this study, both pathogens correlated positively with increased temperature and negatively with increased precipitation, as reported in previous studies (58, 59). *Cladosporium* spp. and *Alternaria* spp. tend to occur more abundantly under dry and warm weather conditions (60). The present work confirmed their positive correlation with warmer and drier conditions and showed a lower frequency in the northernmost geographical location. A future climate including warmer and drier summers could increase the frequency of these fungal pathogens and potentially favor their increase in more northern locations. Studies on the airborne spread over time would allow detecting changes in dispersal patterns in the forest nurseries, which is important to predict the infection risk.

In the current study, *S. polyspora* was found in all four forest nurseries and showed a weak positive correlation with temperature, while there was no significant correlation with precipitation. However, a seasonal variation in the occurrence patterns was confirmed by the fluctuating spore loads. *Sydowia polyspora*, often associated with conifers as a foliar endophyte or opportunistic pathogen, is known to cause reduced seed germination or damage among young seedlings in forest nurseries (23, 61). However, the impact of airborne dispersion of *S. polyspora* on the disease rate in the forest nurseries is yet to be investigated.

The rust fungus *M. populnea* infects *Pinus* spp. and alternate with *Populus tremula* in Northern Europe (62). Basidiospores spread during early summer and can cause sporadic infections of *P. sylvestris* shoots in forest nurseries (4). In this study, regional differences in *M. populnea* were reflected by a higher spore load in the northern nurseries. In addition, *M. populnea* was found to be positively correlated with mean temperature and negatively correlated with mean precipitation. Once infected, *P. sylvestris* can spread aeciospores to the alternating host, on which urediniospores are produced, that can cause re-infection of the alternate host. Aeciospores and urediniospores disperse in late summer and early autumn under warm and dry weather (62). The observed pattern in this study is likely the presence of the different spore types.

Even though not among the more abundant fungal OTUs, *D. sapinea* was detected in this study. *Diplodia sapinea* is an opportunistic and latent pathogen that can cause infection in seeds and seedlings of conifer tree species (35, 63, 64), as well as in mature trees (65). No clear correlation with mean temperature or mean precipitation nor any difference between years or nurseries could be reported, which could be because of the overall low abundance of this fungus in this study.

*Thekopsora areolata* infects mainly *P. abies* cones and can be economically important to seed production (66) but can also infect and cause stem lesions and deformation in nursery-grown seedlings (67). In this study, *T. areolata* was positively correlated with mean temperature and negatively correlated with mean precipitation, showing high peaks early in the growing season. However, the risk of infection in the nursery-grown seedlings is still unclear. Another fungal pathogen detected in this study was *M. betulinum*, a rust fungus infecting *Betula* spp. and causing birch rust in forest nurseries (4). However, the occurrence of *M. betulinum* was not found to correlate with either temperature or precipitation, even though regional differences were observed with higher occurrences in the most northern nursery. The interest in cultivating birch seedlings has increased in recent years, showing that the spore deposition of *M. betulinum* in Swedish forest nurseries will be important for future disease management.

Whether or not observed patterns in spore loads of pathogenic fungi correspond to high or low levels of disease incidences could not be determined, as general disease management strategies, including biological control and fungicide treatments, were used in the studied nurseries. Disease development in nursery-grown tree seedlings is often stress-related. Thus, environmental factors (e.g., drought and frost) or management practices can be important in determining if seedlings will be infected or not, even in the presence of fungal pathogens (23).

### Limitations of the study

The deposited spore loads of fungal pathogens were estimated using relative abundance data and the quantification of the ITS copy number. The ITS copy number varies among fungal species, and this estimation is not precise (68). However, the present study investigated patterns within individual species and did not make any comparisons between species. Therefore, this study provided overall trends for specific pathogens, even though the estimated abundances were not exact. Moreover, for the individual pathogens, the observed variation in the study is several magnitudes larger than the copy number differences found between species. In addition, the sampled spore filters could contain propagules other than spores (e.g., mycelia fragments from germinated spores), contributing to the ITS copy number.

Metabarcoding is a cost-efficient and powerful tool for analyzing fungal communities. However, DNA extraction method, PCR, and sequencing biases (e.g., the choice of primers or sequencing technology) can influence the taxonomic composition and the representation of fungi, as some species might be underrepresented in the community (69). Moreover, the passive spore traps used as a proxy for the realized spore deposition probably capture slightly different fungal communities compared to active spore traps (i.e., the potential spore deposition). For example, the relative importance of vegetation were found to be higher for passive spore traps than for active spore traps (13). Redondo et al. (13) suggested that different spore traps capture species with different dispersal abilities. Thus, putative local taxa might be dominating the fungal communities in the present study, and species with longer dispersal distances could be less represented.

### Implications for airborne disease management

The results in this study showed temporal variations in deposited spore loads of nursery pathogens. The strong shift of the fungal community composition following the growing season suggests that the composition of species changes over time, also shown by the fluctuated occurrence of common species. The airborne presence and abundance of fungi with a strong association to a specific host plant are probably reflected by both the presence and the growth of their host species and favorable weather conditions (14, 15, 70). In contrast, the presence of fungi with a broad host range, which is expected to occur throughout the growing season, is likely to be more influenced by changes in weather conditions (e.g., *Botrytis* spp) (16). The temporal and local variations in this study further indicate that the forest nurseries could expect the risks for fungal infection to differ between the nurseries. In forest nurseries, fungal infections are primarily prevented by cultural control measures, such as removing infected seedlings or reducing moisture among the densely cultivated seedlings. By applying control measures based on decision support systems, the nurseries could increase the accuracy in risk prediction of fungal infections, followed by more precise fungicide applications aligned with the integrated pest management strategy enforced by the EU legislation. Furthermore, an integrated support system could heavily reduce the use of fungicidal treatments compared to calendar-based strategies (71). However, models suitable for tree seedling production are still lacking, and current research on disease models mostly concerns crop and fruit production (e.g., wheat, grapes, potatoes, and apples) (72). Developing accurate forecasting systems requires sufficient knowledge of the relationship between a pathogen and the host plant and their interaction over time and space in a given environment (73). This study provides insight into the dispersal of significant nursery pathogens and their occurrence in Swedish forest nurseries. However, further research on specific nursery pathogens and their interaction with tree seedlings over several seasons is essential before a support system can be implemented in forest nurseries.

### Conclusion

This study provides new knowledge on the airborne dispersal of important nursery pathogens and highlights the influence of seasonality on the temporal dynamics of airborne fungi. Our results suggest strong temporal shifts of airborne fungal diversity and community composition within the growing seasons of forest nurseries, irrespective of the nursery location. The airborne communities included high abundances of important fungal nursery pathogens, with individual temporal and spatial variations. The commonly known nursery pathogen *B. cinerea* did not occur differently between nursery locations or years, whereas *Alternaria* sp. and *Cladosporium* sp. had a lower occurrence in the northmost location. In contrast, *M. betulinum* had the highest occurrence in the north. In general, potential nursery pathogens correlated positively with increased temperature and negatively with increased precipitation. The temperature showed stronger correlations to deposited spore loads than precipitation. This was strongest expressed for *Cladosporium* sp., *Alternaria* sp., and *M. populnea*, which shows a high availability of propagules during warm and dry periods. The seasonal variation should be considered for the development of a local decision support system in forest nurseries, with local adaptations to specific fungal pathogens.

## Data Availability

Sequences of fungal OTUs are available in the GenBank database under accession number KIFZ00000000.

## SUPPLEMENTAL MATERIAL

Supplemental material aem.01306-24-s0001.docx Tables S1 and S3 to S9; Figures S1 and S2.

Table S2 aem.01306-24-s0002.xlsx Final OTU table of 2,152 fungal OTUs.

ASM does not own the copyrights to Supplemental Material that may be linked to, or accessed through, an article. The authors have granted ASM a non-exclusive, world-wide license to publish the Supplemental Material files. Please contact the corresponding author directly for reuse.
