# Supplementary material for: Temporal dynamics of airborne fungi in Swedish forest nurseries
Source: Appl Environ Microbiol. 2025 Jan 16;91(2):e01306-24. doi: 10.1128/aem.01306-24 (PMC11837532; doi:10.1128/aem.01306-24)
Supplement: Supplemental material — Tables S1 and S3 to S9; Figures S1 and S2. [file aem.01306-24-s0001.docx]

| Week | | 18 | 19 | | 20 | | 21 | | 22 | | 23 | | 24 | | 25 | | 26 | | 27 | 28 | | 29 | | 30 | | 31 | | 32 | | 33 | | 34 | | 35 | | 36 | | 37 | | 38 | | 39 | | 40 | | 41 | | 42 | | 43 | | 44 | | 45 | | 46 | | No. of samples | |
| --- | --- | --- | --- | --- | --- | --- | --- | --- | --- | --- | --- | --- | --- | --- | --- | --- | --- | --- | --- | --- | --- | --- | --- | --- | --- | --- | --- | --- | --- | --- | --- | --- | --- | --- | --- | --- | --- | --- | --- | --- | --- | --- | --- | --- | --- | --- | --- | --- | --- | --- | --- | --- | --- | --- | --- | --- | --- | --- | --- |
| 2020 | Trekanten | x | x | | x | | x | | x | | x | | x | | x | | x | | x | x | | x | | x | | x | | x | | x | | x | | x | | x | | x | | x | | x | | x | | x | | x | | x | | x | | x | | x | | 87 | |
|  | Vibytorp | x | x | | x | | x | | (-) | | x | | x | | x | | x | | x | x | | x | | x | | x | | x | | x | | x | | x | | x | | x | | x | | x | | x | | x | | x | | x | | x | | x | | (-) | | 81 | |
|  | Lugnet | x | x | | x | | x | | x | | x | | x | | x | | x | | x | x | | x | | x | | x | | x | | x | | x | | x | | x | | x | | x | | x | | x | | x | | x | | x | | x | | x | | x | | 87 | |
|  |  |  |  | |  | |  | |  | |  | |  | |  | |  | |  |  | |  | |  | |  | |  | |  | |  | |  | |  | |  | |  | |  | |  | |  | |  | |  | |  | |  | |  | |  | |
|  | Week | 18 | 19 | | 20 | | 21 | | 22 | | 23 | | 24 | | 25 | | 26 | | 27 | 28 | | 29 | | 30 | | 31 | | 32 | | 33 | | 34 | | 35 | | 36 | | 37 | | 38 | | 39 | | 40 | | 41 | | 42 | | 43 | | 44 | | 45 | | 46 | |  | |
| 2021 | Trekanten | x | x | | x | | x | | x | | x | | x | | x | | x | | x | x | | x | | (-) | | x | | x | | x | | x | | x | | x | | x | | x | | x | | x | | x | | x | | x | | x | | x | | x | | 84 | |
|  | Vibytorp | (-) | x | | x | | x | | x | | x | | x | | x | | x | | x | x | | x | | (-) | | (-) | | (-) | | (-) | | (-) | | (-) | | (-) | | x | | x | | x | | x | | x | | x | | (-) | | x | | x | | x | | 60 | |
|  | Lugnet | x | x | | x | | x | | x | | x | | x | | x | | x | | x | x | | x | | x | | (-) | | x | | x | | x | | x | | x | | x | | x | | x | | x | | (-) | | x | | x | | x | | x | | x | | 81 | |
|  | Stakheden | x | x | | x | | x | | x | | x | | x | | x | | x | | x | x | | x | | x | | x | | x | | x | | x | | x | | x | | x | | x | | x | | x | | x | | x | | x | | x | | x | | x | | 87 | |
| Total no. of samples | |  | |  | |  | |  | |  | |  | |  | |  | |  | | |  | |  | |  | |  | |  | |  | |  | |  | |  | |  | |  | |  | |  | |  | |  | |  | |  | |  | |  | | 567 |
| Note: weeks that were not sampled or missing data are indicated with (-). | | | | | | | | | | | | | | | | | | | | | | | | | | | | | | | | | | | | | | | | | | | | | | | | | | | | | | | | | | | |

Table S1. Years and weeks of spore collection from passive spore traps (n=3) placed at Trekanten, Vibytorp, Lugnet, and Stakheden forest nurseries, respectively.

Table S3. Effects of year, nursery, week, and the interaction of year and week on the no. of fungal OTUs, the Shannon diversity index, and the Simpson's evenness index.

| **Variable** | | **Chisq** | **df** | **Pr(>Chisq)** |  |
| --- | --- | --- | --- | --- | --- |
| No. of fungal OTUs |  |  |  |  |  |
| (Intercept) | | 532.9 | 1 | <0.001*** |  |
| Tot.reads | | 59.0 | 1 | <0.0001*** |  |
| Year | | 6.2 | 1 | <0.05* |  |
| Nursery | | 14.1 | 3 | <0.01** |  |
| Week | | 140.1 | 28 | <0.001*** |  |
| Year:Week | | 58.5 | 28 | <0.001*** |  |
| Shannon diversity index | |  |  |  |  |
| (Intercept) | | 347.6 | 1 | < 0.001*** |  |
| Tot.reads | | 3.7 | 1 | 0.06 |  |
| Year | | 2.0 | 1 | 0.17 |  |
| Nursery | | 26.7 | 3 | **<** 0.001*** |  |
| Week | | 231.0 | 28 | < 0.001*** |  |
| Year:Week | | 71.2 | 28 | < 0.001*** |  |
| Simpson’s evenness index |  |  |  |  |  |
| (Intercept) | | 94.5 | 1 | < 0.001*** |  |
| Tot.reads | | 0.2 | 1 | 0.66 |  |
| Year | | 6.0 | 1 | <0.05* |  |
| Nursery | | 14.7 | 3 | < 0.01** |  |
| Week | | 179.5 | 28 | < 0.001*** |  |
| Year:Week | | 78.0 | 28 | < 0.001*** |  |
| Note: analyses are based on non-rarefied datasets adjusted using a box-cox transformation in general mixed-effects models. Significant values are indicated with *(*p* < 0.05), **(*p* < 0.01), and ***(*p* < 0.001). | | | | | |

Table S4. Effects of nursery and week for each year, separately, on a) Shannon diversity index and b) Simpson's evenness index.

|  | **2020** | | | | | | **2021** | | | | | | | |
| --- | --- | --- | --- | --- | --- | --- | --- | --- | --- | --- | --- | --- | --- | --- |
|  | **Chisq** | | **df** | | **Pr(>Cisq)** | | **Chisq** | | **df** | | **Pr(>Cisq)** | | | |
| a) No. of fungal OTUs | | | | | | | | | | | | |  |  |
| (Intercept) | | 221.0 | | 1 | | <0.001*** | | 392.3 | | 1 | | <0.001*** | |  |
| Tot.reads | | 13.5 | | 1 | | <0.001*** | | 49.8 | | 1 | | <0.001*** | |  |
| Nursery | | 4.6 | | 2 | | 0.10 | | 10.2 | | 3 | | <0.05* | |  |
| Week | | 105.7 | | 28 | | <0.001*** | | 90.7 | | 28 | | <0.001*** | |  |
| b) Shannon diversity index | |  | |  | |  | |  | |  | |  | |  |
| (Intercept) | | 158.6 | | 1 | | <0.001*** | | 182.1 | | 1 | | <0.001*** | |  |
| Tot.reads | | 0.2 | | 1 | | 0.69 | | 4.0 | | 1 | | <0.05* | |  |
| Nursery | | 2.9 | | 2 | | 0.23 | | 22.4 | | 3 | | <0.001*** | |  |
| Week | | 194.8 | | 28 | | <0.001*** | | 115.6 | | 28 | | <0.001*** | |  |
| c) Simpson's evenness index | | | | | | | | | | | | |  |  |
| (Intercept) | | 34.8 | | 1 | | <0.001*** | | 60.7 | | 1 | | <0.001*** | |  |
| Tot.reads | | 0.08 | | 1 | | 0.77 | | 0.65 | | 1 | | 0.421 | |  |
| Nursery | | 1.8 | | 2 | | 0.41 | | 18.8 | | 3 | | <0.001*** | |  |
| Week | | 139.0 | | 28 | | <0.001*** | | 115.4 | | 28 | | <0.001*** | |  |
| Note: Note: analyses are based on box-cox transformed datasets in general mixed-effects models. Significant values are indicated with *(*p* < 0.05), **(*p* < 0.01), and ***(*p* < 0.001) | | | | | | | | | | | | | | |

Table S5. A pairwise comparison of the estimated marginal means between nurseries for Shannon diversity index and Simpson’s evenness index, respectively, in 2021.

|  | **Pairs of nurseries** | **Estimate** | **SE** |  | **df** | **t.ratio** | **p.value** |
| --- | --- | --- | --- | --- | --- | --- | --- |
| No. of fungal OTUs | Trekanten vs Vibytorp | 0.029 | 0.092 |  | 12.0 | 0.313 | 0.989 |
|  | Trekanten vs Lugnet | 0.103 | 0.083 |  | 7.8 | 1.243 | 0.620 |
|  | Trekanten vs Stakheden | 0.217 | 0.081 |  | 7.1 | 2.682 | 0.112 |
|  | Vibytorp vs Lugnet | 0.132 | 0.092 |  | 11.9 | 1.425 | 0.510 |
|  | Vibytorp vs Stakheden | 0.246 | 0.091 |  | 11.5 | 2.686 | 0.083 |
|  | Lugnet vs Stakheden | -0.114 | 0.082 |  | 7.3 | -1.394 | 0.539 |
| Shannon diversity index | Trekanten vs Vibytorp | 0.143 | 0.104 |  | 12.0 | 1.368 | 0.541 |
|  | Trekanten vs Lugnet | 0.229 | 0.095 |  | 7.8 | 2.423 | 0.151 |
|  | Trekanten vs Stakheden | 0.280 | 0.092 |  | 7.1 | 3.032 | 0.071 |
|  | Vibytorp vs Lugnet | 0.372 | 0.105 |  | 11.9 | 3.530 | **0.019** |
|  | Vibytorp vs Stakheden | 0.423 | 0.104 |  | 11.5 | 4.050 | **0.008** |
|  | Lugnet vs Stakheden | -0.051 | 0.093 |  | 7.3 | -0.545 | 0.945 |
| Simpson’s evenness index | Trekanten vs Vibytorp | 0.014 | 0.013 |  | 12.0 | 1.137 | 0.675 |
|  | Trekanten vs Lugnet | 0.030 | 0.011 |  | 7.8 | 2.682 | 0.106 |
|  | Trekanten vs Stakheden | 0.028 | 0.011 |  | 7.1 | 2.523 | 0.139 |
|  | Vibytorp vs Lugnet | 0.044 | 0.013 |  | 11.9 | 3.533 | **0.019** |
|  | Vibytorp vs Stakheden | 0.042 | 0.013 |  | 11.5 | 3.370 | **0.026** |
|  | Lugnet vs Stakheden | 0.002 | 0.011 |  | 7.3 | 0.219 | 0.996 |
| Note: P-values are adjusted with Tukey’s method of comparing a family of four estimates and significant differences (*p* < 0.05) are indicated in bold. | | | | | | | |

Table S6. Permutational multivariate analysis of variance (PERMANOVA) of the fungal community composition from spore traps collected in 2020 and 2021 at Trekanten, Vibytorp, Lugnet, and Stakheden forest nurseries.

| **Variable** | **Df** | **Sum.Sq** |  | **R^2^** | **F** | **Pr(>F)** |
| --- | --- | --- | --- | --- | --- | --- |
| Tot. reads | 1 | 0.674 |  | 0.004 | 3.272 | **0.001** |
| Year | 1 | 3.002 |  | 0.019 | 14.566 | **0.001** |
| Nursery | 3 | 5.652 |  | 0.035 | 9.141 | **0.001** |
| Week | 28 | 34.109 |  | 0.212 | 5.911 | **0.001** |
| Year:Week | 28 | 13.323 |  | 0.083 | 2.309 | **0.001** |
| Residuals | 505 | 104.083 |  | 0.648 |  |  |
| Total | 566 | 160.844 |  | 1.000 |  |  |

Note: Significant differences (*p* < 0.001) are indicated in bold.

Table S7. Effects of year, nursery, week, and the interaction of year and week, on the ITS copy number of nursery fungal pathogens.

| Fungal OTU | **Year** | **Nursery** | **Week** | **Year:Week** |  |
| --- | --- | --- | --- | --- | --- |
| *Cladosporium* sp. | Chisq=21.8*** | Chisq=13.3** | Chisq=170.8*** | Chisq=51.1** |  |
| *Alternaria* sp. | Chisq=15.0*** | Chisq=14.7** | Chisq=199.9*** | Chisq=67.6*** |  |
| *Thekopsora areolata* | Chisq=0.7 | Chisq=54.4*** | Chisq=295.0*** | Chisq=70.1*** |  |
| *Botrytis cinerea* | Chisq=0.05 | Chisq=3.0 | Chisq=56.9** | Chisq=67.1*** |  |
| *Sydowia polyspora* | Chisq=17.9*** | Chisq=2.5 | Chisq=133.5*** | Chisq=51.9** |  |
| *Melampsora populnea* | Chisq=0.7 | Chisq=15.0** | Chisq=245.4*** | Chisq=58.4*** |  |
| *Melampsoridium betulinum* | Chisq=13.6*** | Chisq=16.0** | Chisq=190.4*** | Chisq=92.1*** |  |
| *Diplodia sapinea* | Chisq=2.4 | Chisq=9.4* | Chisq=143.9*** | Chisq=81.8*** |  |
| Note: analyses are based on box-cox transformed datasets in general mixed-effects models. Significant values are indicated with *(*p* < 0.05), **(*p* < 0.01), and ***(*p* < 0.001). | | | | | |

Table S8. A pairwise comparison between forest nurseries of the estimated marginal means of ITS copy number for nursery pathogens in 2020.

|  | **Pairs of nurseries** | **Estimate** | **SE** | **df** | **t.ratio** | **p.value** |
| --- | --- | --- | --- | --- | --- | --- |
| *Cladosporium* sp. | Trekanten vs Vibytorp | -1.17 | 0.758 | 223 | -1.539 | 0.275 |
|  | Trekanten vs Lugnet | 1.88 | 0.740 | 223 | 2.536 | **0.032** |
|  | Vibytorp vs Lugnet | 0.71 | 0.758 | 223 | 0.936 | 0.618 |
| *Alternaria* sp. | Trekanten vs Vibytorp | -0.22 | 0.747 | 223 | -0.292 | 0.954 |
|  | Trekanten vs Lugnet | 1.57 | 0.729 | 223 | 2.147 | 0.083 |
|  | Vibytorp vs Lugnet | 1.35 | 0.747 | 223 | 1.804 | 0.171 |
| *T. areolata* | Trekanten vs Vibytorp | -1.01 | 0.599 | 223 | -1.692 | 0.211 |
|  | Trekanten vs Lugnet | 2.45 | 0.584 | 223 | 4.190 | **<0.001** |
|  | Vibytorp vs Lugnet | 1.44 | 0.599 | 223 | 2.397 | **0.046** |
| *M. populnea* | Trekanten vs Vibytorp | -0.07 | 0.619 | 223 | -0.116 | 0.993 |
|  | Trekanten vs Lugnet | 1.95 | 0.604 | 223 | 3.228 | **0.004** |
|  | Vibytorp vs Lugnet | 1.88 | 0.619 | 223 | 3.035 | **0.008** |
| *M. betulinum* | Trekanten vs Vibytorp | -0.325 | 1.34 | 223 | -0.242 | 0.968 |
|  | Trekanten vs Lugnet | 0.66 | 1.31 | 223 | 0.503 | 0.870 |
|  | Vibytorp vs Lugnet | 0.335 | 1.34 | 223 | 0.249 | 0.966 |
| *D. sapinea* | Trekanten vs Vibytorp | 6.39 | 2.97 | 223 | 2.151 | 0.082 |
|  | Trekanten vs Lugnet | -2.01 | 2.90 | 223 | -0.692 | 0.769 |
|  | Vibytorp vs Lugnet | 4.38 | 2.97 | 223 | 1.476 | 0.305 |
| Note: P-values are adjusted with Tukey’s method of comparing a family of four estimates and significant differences (*p* < 0.05) are indicated in bold. | | | | | | |

Table S9. A pairwise comparison between forest nurseries of the estimated marginal means of ITS copy number for nursery pathogens in 2021.

|  | **Pairs of nurseries** | **Estimate** | **SE** | **df** | **t.ratio** | **p.value** |
| --- | --- | --- | --- | --- | --- | --- |
| *Cladosporium* sp. | Trekanten vs Vibytorp | 0.17 | 0.922 | 279 | 0.184 | 0.998 |
|  | Trekanten vs Lugnet | -0.04 | 0.836 | 279 | -0.050 | 1.000 |
|  | Trekanten vs Stakheden | -2.78 | 0.817 | 279 | -3.407 | **0.004** |
|  | Vibytorp vs Lugnet | 0.13 | 0.933 | 279 | 0.137 | 0.999 |
|  | Vibytorp vs Stakheden | -2.61 | 0.919 | 279 | -2.844 | **0.025** |
|  | Lugnet vs Stakheden | 2.74 | 0.825 | 279 | 3.323 | **0.006** |
| *Alternaria* sp. | Trekanten vs Vibytorp | 0.71 | 0.918 | 279 | 0.774 | 0.866 |
|  | Trekanten vs Lugnet | -0.16 | 0.833 | 279 | -0.194 | 0.997 |
|  | Trekanten vs Stakheden | -2.97 | 0.814 | 279 | -3.652 | **0.002** |
|  | Vibytorp vs Lugnet | 0.55 | 0.929 | 279 | 0.592 | 0.935 |
|  | Vibytorp vs Stakheden | -2.26 | 0.915 | 279 | -2.247 | 0.067 |
|  | Lugnet vs Stakheden | 2.81 | 0.822 | 279 | 3.420 | **0.004** |
| *T. areolata* | Trekanten vs Vibytorp | -0.18 | 0.756 | 279 | -0.234 | 0.996 |
|  | Trekanten vs Lugnet | 1.98 | 0.686 | 279 | 2.890 | **0.022** |
|  | Trekanten vs Stakheden | 3.59 | 0.670 | 279 | 5.356 | **<0.001** |
|  | Vibytorp vs Lugnet | 1.80 | 0.765 | 279 | 2.359 | 0.088 |
|  | Vibytorp vs Stakheden | 3.41 | 0.754 | 279 | 4.527 | **<0.001** |
|  | Lugnet vs Stakheden | -1.61 | 0.676 | 279 | -2.375 | 0.084 |
| *M. populnea* | Trekanten vs Vibytorp | -0.34 | 0.765 | 279 | -0.438 | 0.972 |
|  | Trekanten vs Lugnet | 1.94 | 0.693 | 279 | 2.798 | **0.028** |
|  | Trekanten vs Stakheden | 1.31 | 0.677 | 279 | 1.936 | 0.215 |
|  | Vibytorp vs Lugnet | 1.61 | 0.774 | 279 | 2.075 | 0.164 |
|  | Vibytorp vs Stakheden | 0.98 | 0.762 | 279 | 1.282 | 0.575 |
|  | Lugnet vs Stakheden | 0.63 | 0.684 | 279 | 0.919 | 0.795 |
| *M. betulinum* | Trekanten vs Vibytorp | 0.01 | 0.732 | 279 | 0.020 | 1.000 |
|  | Trekanten vs Lugnet | 0.58 | 0.664 | 279 | 0.880 | 0.815 |
|  | Trekanten vs Stakheden | 2.21 | 0.649 | 279 | 3.408 | **0.004** |
|  | Vibytorp vs Lugnet | 0.60 | 0.741 | 279 | 0.809 | 0.850 |
|  | Vibytorp vs Stakheden | 2.22 | 0.729 | 279 | 3.050 | **0.013** |
|  | Lugnet vs Stakheden | -1.63 | 0.655 | 279 | -2.483 | 0.065 |
| *D. sapinea* | Trekanten vs Vibytorp | 18.67 | 19.3 | 279 | 0.967 | 0.768 |
|  | Trekanten vs Lugnet | -21.54 | 17.5 | 279 | -1.231 | 0.607 |
|  | Trekanten vs Stakheden | -41.86 | 17.1 | 279 | -2.449 | 0.071 |
|  | Vibytorp vs Lugnet | -2.88 | 19.5 | 279 | -0.147 | 0.999 |
|  | Vibytorp vs Stakheden | -23.19 | 19.2 | 279 | -1.206 | 0.623 |
|  | Lugnet vs Stakheden | 20.31 | 17.3 | 279 | 1.177 | 0.642 |
| Note: P-values are adjusted with Tukey’s method of comparing a family of four estimates and significant differences (*p* < 0.05) are indicated in bold. | | | | | | |


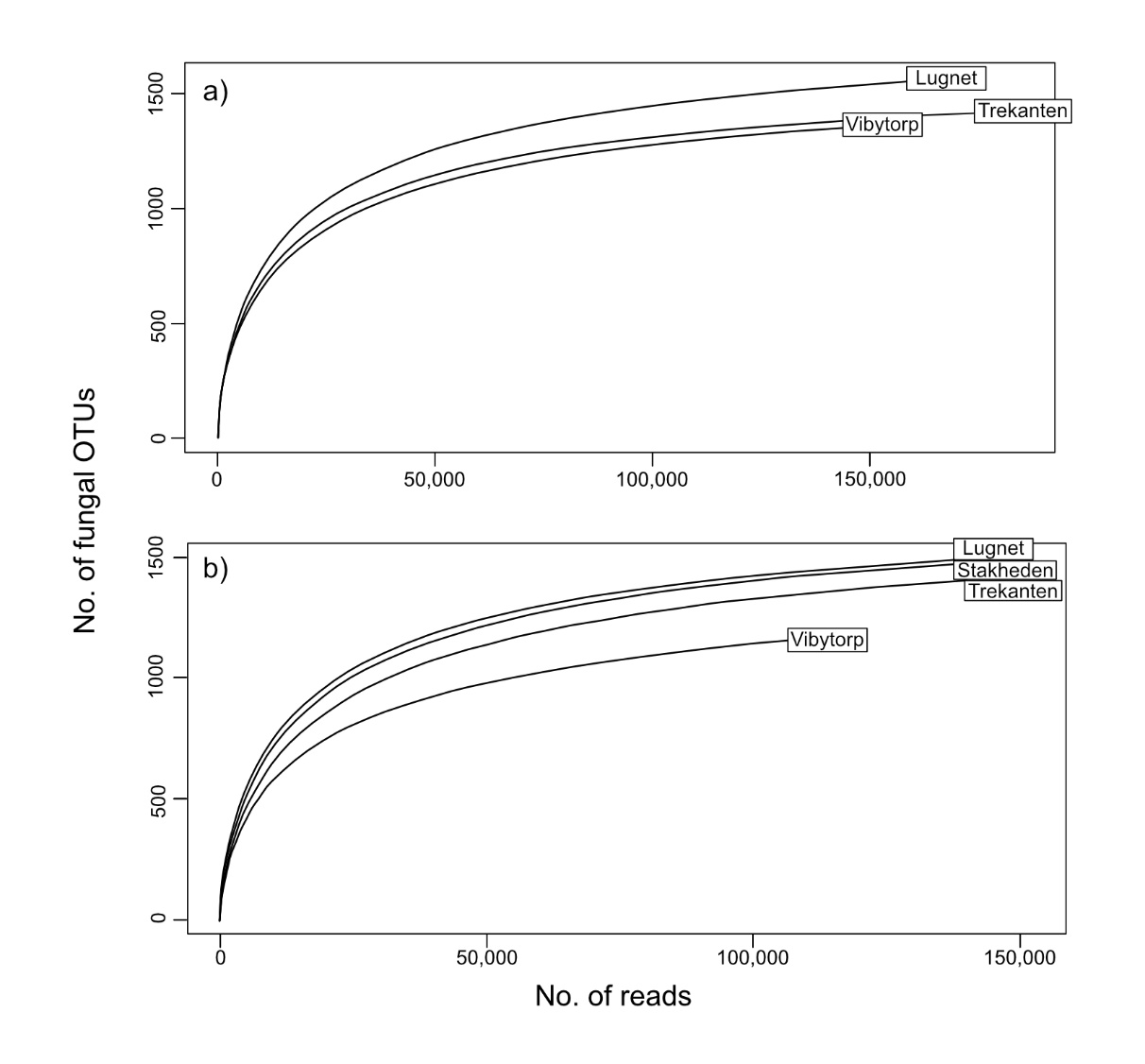


Figure S1. Rarefaction curves showing the relationship between the cumulative number of fungal OTUs and the number of sequences for each sample in four forest nurseries in a) 2020 and b) 2021.


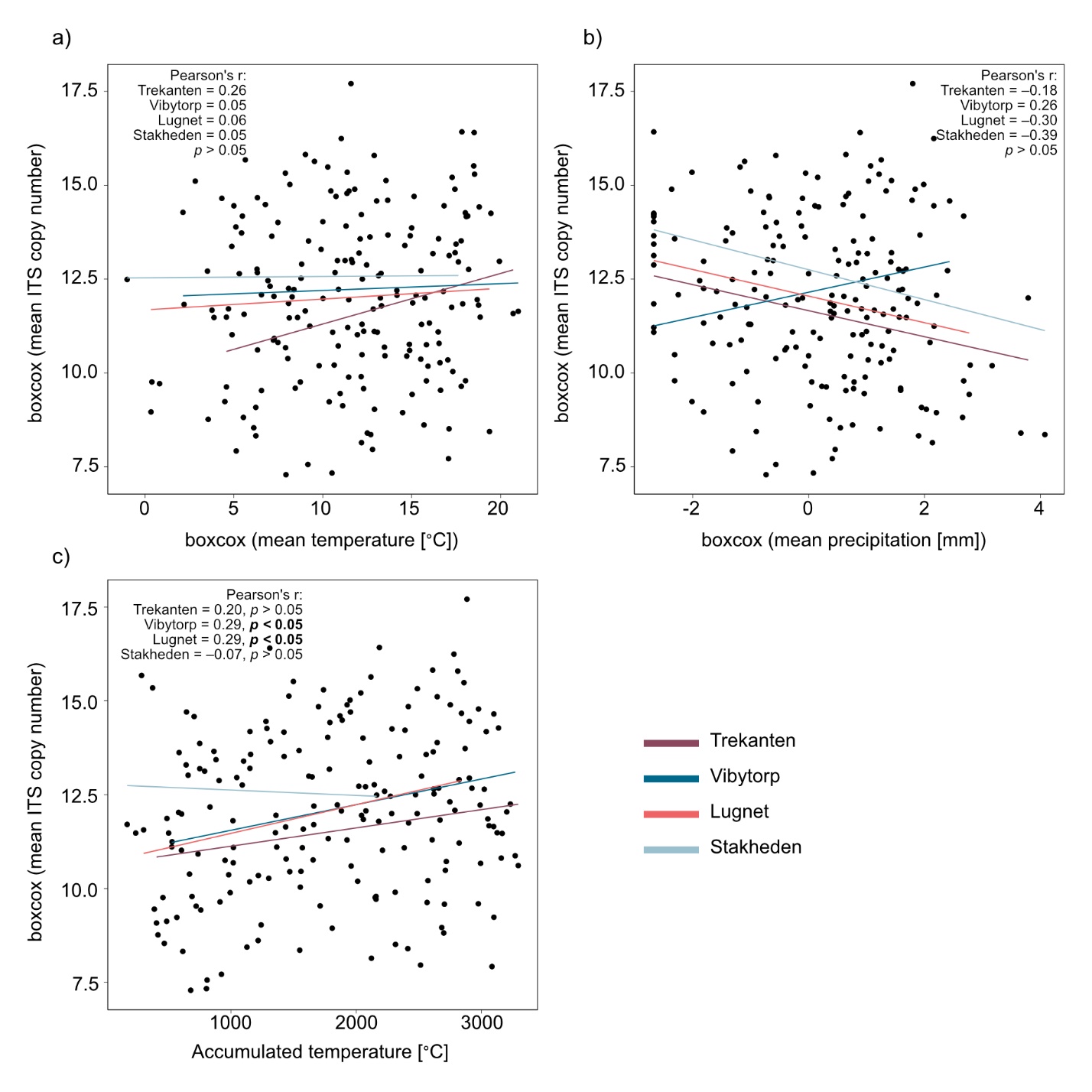


Figure S2. Correlation between ITS copy number obtained from spore traps and a) mean temperature, b) mean precipitation or c) accumulated day-temperature in four forest nurseries. Nurseries are indicated by using different colours. Correlation factors are given as Pearson’s r, and significant differences are indicated in bold.
